# Case Report: Takotsubo Syndrome Induced by Severe Anaphylactic Reaction During Anesthesia Induction and Subsequent High-Dose Epinephrine Resuscitation

**DOI:** 10.3389/fcvm.2022.842440

**Published:** 2022-03-17

**Authors:** Jiaojiao Wei, Le Zhang, Xia Ruan, Kai He, Chunhua Yu, Le Shen

**Affiliations:** ^1^Department of Anesthesiology, Peking Union Medical College, Peking Union Medical College Hospital, Chinese Academy of Medicine Science, Beijing, China; ^2^Department of Anesthesiology, Civil Aviation General Hospital, Beijing, China; ^3^State Key Laboratory of Complex Severe and Rare Diseases, Peking Union Medical College Hospital, Beijing, China

**Keywords:** takotsubo syndrome, anaphylaxis, cardiogenic shock, cardiopulmonary resuscitation, epinephrine, extracorporeal membrane oxygenation

## Abstract

Takotsubo syndrome (TTS) is a type of non-ischemic cardiomyopathy characterized by an acute reversible left ventricular dysfunction with typical apical ballooning, usually with subsequent complete recovery. Early diagnosis and prompt treatment are of great essence. Herein, we described a case of TTS of a patient who was scheduled initially for laparoscopic endometrial cancer staging. The 69-year-old woman presented with cardiogenic shock induced by the severe anaphylactic reaction to the antibiotics during anesthesia induction. Cardiopulmonary resuscitation (CPR) was implemented while several boluses of 1 mg epinephrine were injected. After the return of spontaneous circulation, a large number of orange peel-like rash appeared on the head, face, neck, and trunk of the patient. Transesophageal echocardiography (TEE) revealed diffused decreased left ventricular systolic function. Therefore, veno-arterial extracorporeal membrane oxygenation (VA-ECMO) and intra-aortic balloon pump (IABP) were applied in the intensive care unit. Biomarkers like cardiac troponin I (cTnI) subsequently decreased with improved cardiac insufficiency. Finally, the patient was discharged in good condition. This case demonstrated that TTS could be secondary to severe anaphylactic shock and exogenous catecholamines. With the consideration of the reversible condition and predictable recovery of TTS, early vigilance and advanced life support devices should be necessary.

## Introduction

A severe perioperative anaphylactic reaction is a rare, but life-threatening, condition with an incidence of ~1:7,000–10,000. Possible exposures include antibiotics, anesthetics, colloids, disinfectants, latex, and so on (1). Meanwhile, life-threatening hypotension, bronchospasm, and even cardiopulmonary arrest could be the manifestations in most cases of a severe perioperative anaphylactic reaction, because skin signs might often be absent or neglected during general anesthesia. Immediate administration of low-dose epinephrine is the mainstay of severe anaphylactic reaction treatment. If cardiopulmonary arrest occurred, advanced life support, including cardiopulmonary resuscitation (CPR) and 1 mg boluses of epinephrine, are necessary. Either severe anaphylactic reaction or high-dose epinephrine administration could activate the immune system and result in stress and inflammation storms, which may be associated with significant cardiovascular complications.

Takotsubo syndrome (TTS), also known as Takotsubo cardiomyopathy or stress cardiomyopathy, is a severe syndrome characterized by acute transient left ventricular systolic dysfunction and electrocardiogram changes in the absence of obstructive coronary artery disease (2). TTS could be induced by the acute endogenous release of catecholamines as well as the exogenous administration of large amounts of catecholamines. In addition to catecholamine excess, the pathogenesis of the disorder possibly involves low estradiol level, inflammation response, endothelial dysfunction, and thyroid dysfunction (3–6). Inflammation has been extensively studied, and both myocardial and systemic inflammation are associated with TTS (7). For patients with TTS, higher serum levels of IL-6 and IL-10 at admission could predict an increased risk of adverse events at follow-up (8). Different complications have been reported in TTS, including atrial fibrillation, cardiogenic shock, and thromboembolic events (9, 10). Atrial fibrillation in TTS is not uncommon. The mortality rate is significantly higher in TTS, accompanied by atrial fibrillation, and long-term prognosis decreased in these patients (11). A recent study has shown that patients with TTS suffered more often from thromboembolic events compared with patients with acute coronary syndrome (ACS), and an elevated C-reactive protein (CRP) level might be a predictor of thromboembolic events (12). In this report, we presented a rare case of TTS induced by catecholamine excess and it was accompanied by cardiogenic shock.

## Case Presentation

**Figure 1:**
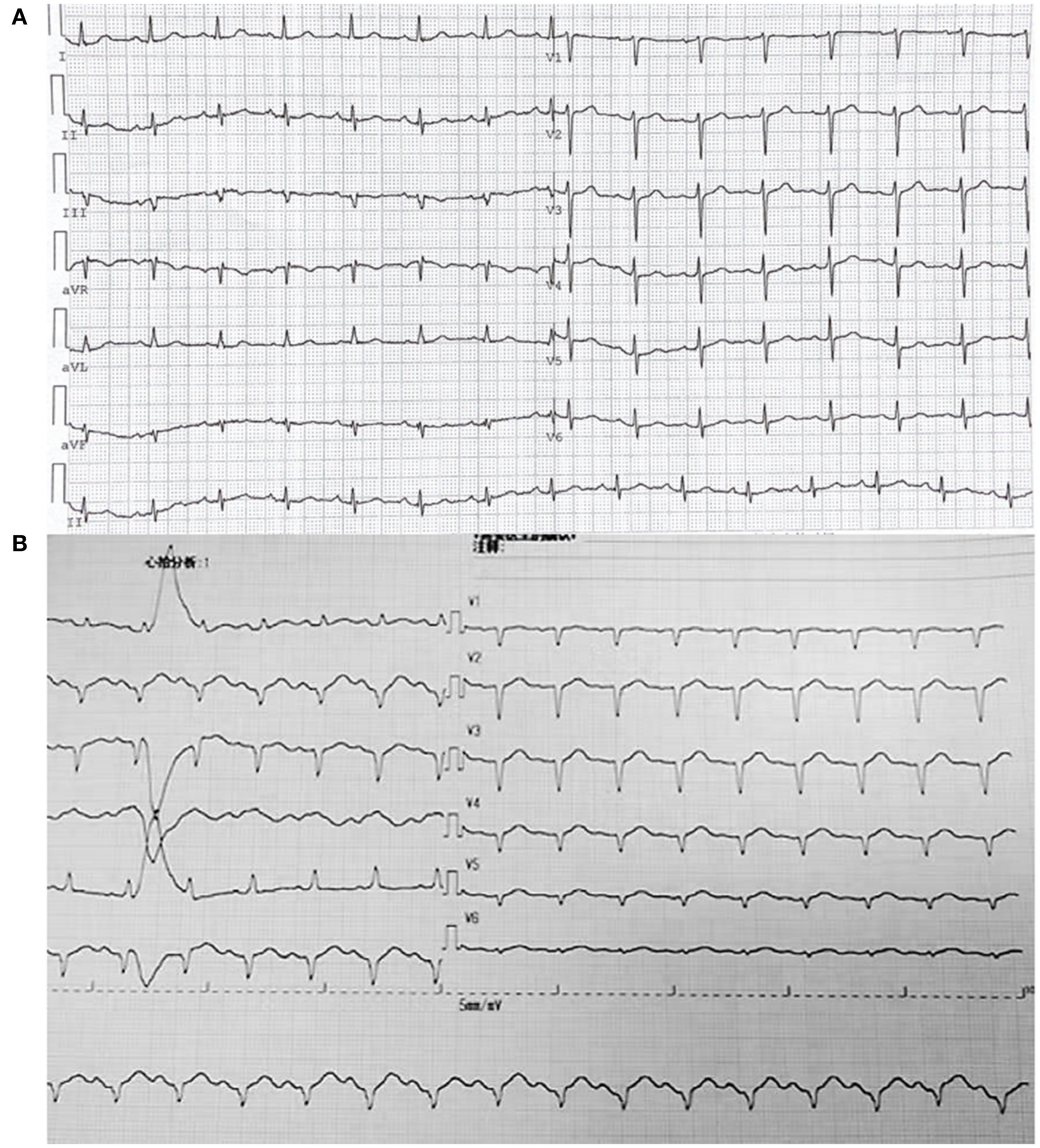
Electrocardiogram **(A)** Before the surgery; Electrocardiogram **(B)** After return of spontaneous circulation (ROSC).

A 69-year-old woman (height: 156 cm; weight: 105 kg) was scheduled for laparoscopic staging of endometrial cancer under general anesthesia. Her past medical history included hypertension (grade III, very high risk), type II diabetes, fatty liver, and breast nodules. She had an allergy to a traditional Chinese medicine, Red Flower Oil. The routine preoperative evaluation showed that her ECG with grade III hypertension and lead aVF was labeled as RS type (Figure 1A), her echocardiography showed reduced left ventricular diastolic function and a left ventricular ejection fraction (LVEF) of 63%, and her coronary artery CT showed mild to moderate coronary atherosclerosis.

Upon arrival in the operating room, routine monitoring, including ECG, noninvasive blood pressure (NBP), and pulse oximetry (SpO_2_), was applied. Lactated Ringer's solution (500 ml) was rapidly infused intravenously during the induction of anesthesia, followed by 1 g of cefmetazole sodium injection. The patient was induced with mask oxygenation, lidocaine (70 mg), fentanyl (150 ug), parecoxib (40 mg), propofol (170 mg), and rocuronium (65 mg IV). Then, endotracheal intubation was performed successfully. Soon after the induction, NBP decreased quickly, and intravenous injection ephedrine or phenylephrine showed no effect. In 10 min, the carotid artery became very weak and could not be palpated; hence, CPR was started while several boluses of 1 mg epinephrine were injected. The NBP returned to 100–130/60–80 mmHg. Within 10 min, a large number of orange peel-like rash appeared on the head, face, neck, and trunk of the patient. To maintain enough circulation, epinephrine was infused with 1 mg/min. Transesophageal echocardiography (TEE) revealed diffused decreased left ventricular systolic function and poor left ventricular lower and posterior wall contraction. Contraction of the posterior interventricular septum, which is the area supplied by the right coronary artery, was generally normal (Supplementary Videos 1, 2). After the return of spontaneous circulation (ROSC), a 12-lead ECG indicated that the R wave of V1-V6 disappeared, and no elevation of the ST-segment was captured (Figure 1B). However, the bilateral pupils of the patient were found dilated to the diameter of 6–7 mm, with weakened light reflectance.

Due to the condition of the patient, the diagnosis was most likely a severe anaphylactic reaction to the antibiotics. After the patient was transferred to the intensive care unit, her spontaneous circulation needed high dose epinephrine, amounting to 0.6–1 μ*g*·kg^−1^·min^−1^, and norepinephrine, amounting to 0.2–0.5 μg·kg^−1^·min^−1^. Levosimendan was used for potential cardiogenic shock. However, the shock status deteriorated with continuously increasing demands of epinephrine and norepinephrine, as well as lactic acid levels. TEE showed reduced systolic function and poor anterior interval contraction. In addition, veno-arterial extracorporeal membrane oxygenation (VA-ECMO) was applied.

On a postoperative day one (POD1), the ECG of the patient manifested II, III, AVF, and V1-V6 leads as QS type, and her lactate was raised to 19.1 mmol/L, blood glucose to 28.7 mmol/ L, WBC to 35.12 × 10^9^/L, and cTnI raised from 11.038 to 27.68 ug/L. Upon these conditions, the diagnosis was most likely TTS. Afterward, the IABP was placed. With the support of VA-ECMO and IABP, epinephrine was discontinued on POD3. The VA-ECMO was removed on POD4. IABP was removed on POD5. cTnI became normal on POD6. The patient was successfully extubated on POD10, followed by the additional one-day non-invasive ventilator. On POD12, norepinephrine was discontinued. On POD14, the patient was transferred back to the general ward, and on POD17, she was discharged in good condition and had continued to use aspirin after discharge.

## Discussion

The reported patient finally recovered successfully from a severe cardiogenic shock due to severe anaphylactic reaction, CPR, and TTS sequentially. TTS is usually caused by the acute endogenous release of catecholamines with emotional or physical triggers. Occasionally, TTS could also be caused by exogenous administration of a large number of catecholamines. TTS always leads to myocardial inflammation and short-term left ventricular insufficiency (13). As an important and necessary protective mechanism, the heart needs to “have a rest” to maintain myocardial integrity that displayed as symmetric regional wall motion abnormalities (RWMAs).

However, TTS is generally a benign and reversible condition with predictable recovery, and the RWMAs are usually transient. During the TTS progressing, excessive endogenous or exogenous epinephrine triggers the conversion of the β2-epinephrine receptor from GS to GI coupling, resulting in a negative inotropic response that limits the severity of acute myocardial injury caused by catecholamines. Secondarily, the activation of the phosphoinositol 3-kinase/protein kinase B (Akt) pathway and induced angiogenesis are essential for the improvement of left ventricular function after myocardial injury (14).

**Figure 2:**
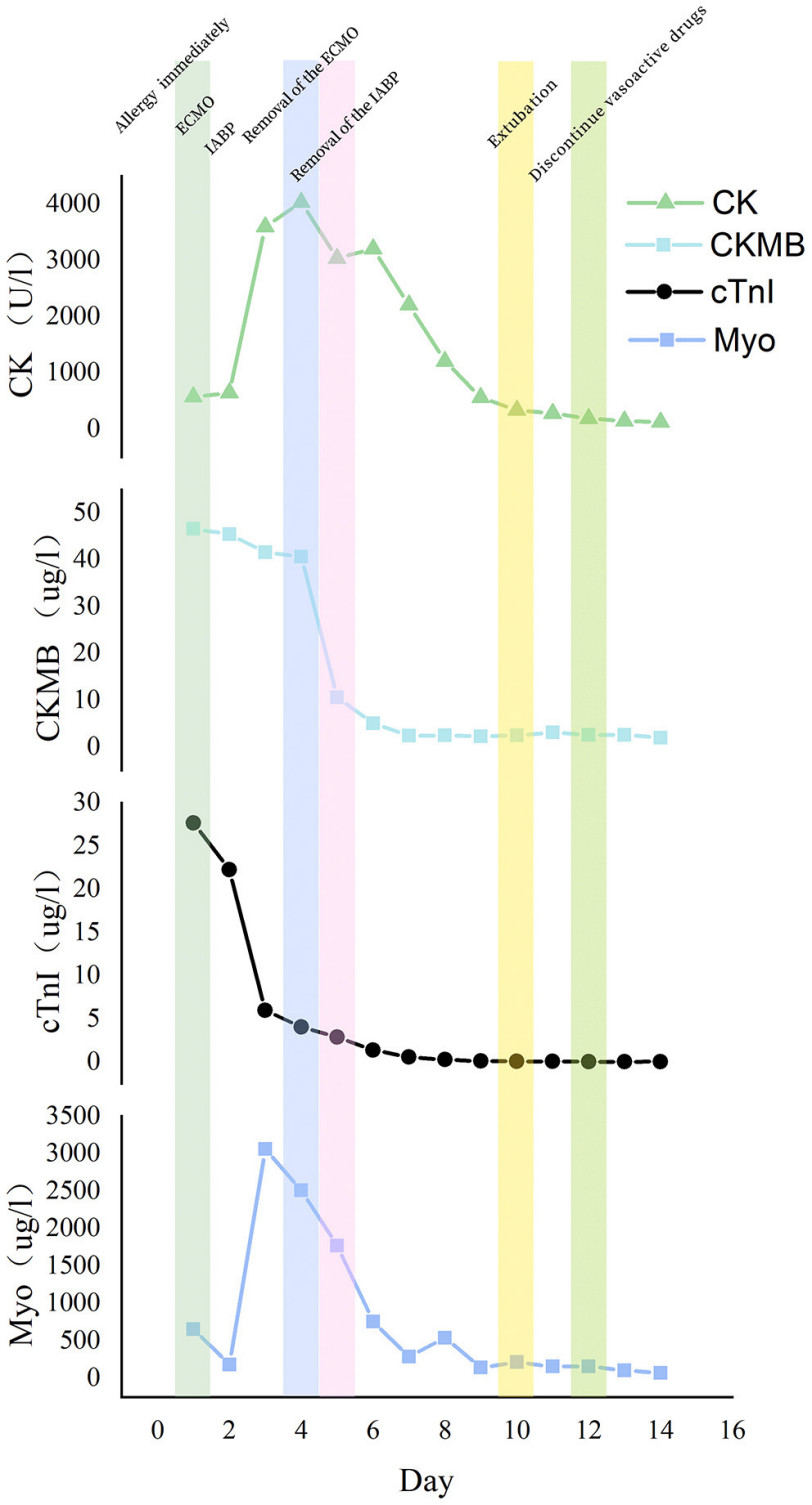
The variation of biomarkers during hospital course. CK, creatine kinase; CKMB, creatine kinase-MB; cTnI, cardiac troponin I; Myo, myoglobin.

The diagnostic algorithm for TTS consists of electrocardiogram, InterTAK Diagnostic Score, biomarkers (markers of myocardial necrosis, B-type natriuretic peptide, and N-terminal prohormone of brain natriuretic peptide), and imagings (coronary angiography and ventriculography, echocardiography, cardiac CT angiography, cardiac MRI, cardiac nuclear imaging, perfusion imaging, metabolic imaging, and sympathetic nervous imaging). This patient received more than 10 mg epinephrine in a short time, which was combined with endogenous catecholamine release during the severe anaphylactic reaction, thereby causing TTS and cardiogenic shock (15). She displayed non-ST-segment elevation, with the InterTAK Diagnostic criteria partially met (Table 1). The biomarkers were all lately reported to be elevated significantly. cTnI and creatine kinase (CK)-MB peaked after applying VA-ECMO, while CK and myoglobin reached the top 2 or 3 days later. These biomarkers dropped as her left ventricular systolic function improved (Figure 2). There is another concern that obesity prompted the risk of perioperative rhabdomyolysis, which is characterized by elevated serum muscle proteins, including CK and myoglobin (16). The theory could explain why CK and myoglobin peaked later than cTnI and decreased with advanced life support, as well as early bedside exercise. Because of low-quality TTE imaging, an immediate point-of-care TEE is of great necessity for the differential diagnosis of such a sudden collapse of the cardiovascular system, especially for the ruling out of acute myocardial infarction. Her left ventricular wall motion abnormalities extend beyond the distribution of a single coronary artery territory, as is featured in TTS (17). As for imaging, the following 4 different TTS forms have been reported to date: apical form, midventricular form, basal form, and rare focal form. This case belongs to the midventricular form according to the manifestation of TEE (18, 19). El-Battrawy et al. reported that the incidence of RV involvement in TTS was 11%. Furthermore, patients with RV involvement had a higher incidence of in-hospital cardiogenic shock, which helped explain the progression of this case (20).

**Table 1:** InterTAK Diagnostic Criteria for the case.

**International Takotsubo Diagnostic Criteria (InterTAK Diagnostic Criteria)**	**This case**
1. Patients show transient left ventricular dysfunction (hypokinesia, akinesia, or dyskinesia) presenting as apical ballooning or midventricular, basal, or focal wall motion abnormalities. Right ventricular involvement can be present. Besides these regional wall motion patterns, transitions between all types can exist. The regional wall motion abnormality usually extends beyond a single epicardial vascular distribution; however, rare cases can exist where the regional wall motion abnormality is present in the subtended myocardial territory of a single coronary artery (focal TTS).b	√
2. An emotional, physical, or combined trigger can precede the takotsubo syndrome event, but this is not obligatory.	√
3. Neurologic disorders (e.g., subarachnoid hemorrhage, stroke/transient ischaemic attack, or seizures) as well as pheochromocytoma may serve as triggers for takotsubo syndrome.	N/A
4. New ECG abnormalities are present (ST-segment elevation, ST-segment depression, T-wave inversion, and QTc prolongation); however, rare cases exist without any ECG changes.	√
5. Levels of cardiac biomarkers (troponin and creatine kinase) are moderately elevated in most cases; significant elevation of brain natriuretic peptide is common.	√
6. Significant coronary artery disease is not a contradiction in takotsubo syndrome.	N/A
7. Patients have no evidence of infectious myocarditis.	√
8. Postmenopausal women are predominantly affected.	√

When TTS is combined with cardiogenic shock, the Ca^2+^ sensitizer levosimendan has been suggested as being used safely and effectively as an alternative inotrope to catecholamine agents. Beta-blockers may also improve left ventricular outflow tract obstruction (LVOTO) but are contraindicated in acute and severe heart failure with low LVEF, hypotension, and bradycardia when the I_f_ channel inhibitor, ivabradine, may be beneficial. The left ventricular assist device (LVAD) and VA-ECMO are effective for patients with TTS during primary pump failure conditions. As a thrombogenic state may arise as a consequence of catecholamine-dependent ventricular dysfunction, platelet activation, and vasoconstriction during the acute phase of TTS, antiplatelet therapy during hospitalization seems to be an appropriate choice for patients with TTS. However, a recent study found that aspirin at discharge did not relate to a reduced risk of major adverse cardiac and cerebrovascular events in patients with TTS (21).

The primary diagnosis of the patient was endometrial cancer. A current study has presented a strong association between TTC and malignant diseases, and the association may be due to a common pathway, namely an individually high catecholaminergic state (22). Either the history of or the current cancer are associated with an increased risk of adverse events in patients with TTS. Therefore, a careful follow-up after an episode of TTS in patients with cancer counts a lot.

## Data Availability Statement

The raw data supporting the conclusions of this article will be made available by the authors, without undue reservation.

## Ethics Statement

Written informed consent was obtained from the individual(s) for the publication of any potentially identifiable images or data included in this article.

## Author Contributions

JW looked after the patient and wrote the report. LZ was involved in editing and writing assistance. XR assisted in the CPR of the case. KH and CY did the TEE examination for the case. LS supervised the patient's anesthesia and case report writing. All authors have read and approved the final version of the manuscript.

## Funding

This work was supported by the Education Reform Project Foundation for the Central Universities of Peking Union Medical College (2020zlgc0105), the Training Programme Foundation for Excellent Talents in Dongcheng District of Beijing (2019DCT-M-08), and the Non-profit Central Research Institute Fund of Chinese Academy of Medical Sciences (2019XK320018).

## Conflict of Interest

The authors declare that the research was conducted in the absence of any commercial or financial relationships that could be construed as a potential conflict of interest.

## Publisher's Note

All claims expressed in this article are solely those of the authors and do not necessarily represent those of their affiliated organizations, or those of the publisher, the editors and the reviewers. Any product that may be evaluated in this article, or claim that may be made by its manufacturer, is not guaranteed or endorsed by the publisher.
